# Prevalence of frailty in hemodialysis patients and its impact on long-term survival in elderly hemodialysis patients

**DOI:** 10.3389/fmed.2026.1857811

**Published:** 2026-06-01

**Authors:** Shunlai Shang, Liu Yang, Qi Zhang, Wan Dong, Jing Hu, Wanning Jia, Liqian Gao, Yonge Wang, Cheng Zhou, Haifeng Wang, Wenwen He

**Affiliations:** 1 Department of Nephrology, China-Japan Friendship Hospital, Beijing, China; 2 China Institute of Clinical Medical Sciences, China-Japan Friendship Hospital, Beijing, China

**Keywords:** China, frailty, maintenance hemodialysis, mortality risk, older adults, survival analysis

## Abstract

**Background:**

The number of dialysis patients in China is rapidly increasing, with the proportion of older adults aged ≥ 60 years rising, linked to population aging and longer dialysis duration. This study investigated frailty prevalence in Chinese maintenance hemodialysis (MHD) patients, analyzed its status, risk factors and correlation with long-term survival in older dialysis patients to provide evidence for frailty interventions.

**Methods:**

A total of 391 patients were assessed for frailty using the Frailty Screening Scale. Demographic, clinical, and biochemical data were collected, with 24-month follow-up for survival. Multivariate regression and Cox regression models were used for analyses.

**Results:**

The cohort comprised 222 male patients (56.9%), with a mean age of 61 ± 13.9 years and a median dialysis vintage of 4 (2.0, 8.5) years. Patients aged ≥ 60 years accounted for 57.5% of participants. Frailty assessment showed that 85.3% of older patients had varying degrees of frailty, and 52.7% were in the pre-frail status. Increased age and low albumin level were identified as clinical correlates of frailty. During the 24-month follow-up period, a total of 88 deaths occurred. Regression analysis showed that patients under maintenance hemodialysis with frailty syndrome had a 3–5-fold higher mortality risk compared with non-frail individuals, and this association was more prominent in the elderly population aged over 60 years. Compared with patients with frailty syndrome, individuals in the pre-frail stage had markedly reduced mortality (*P* = 0.009, *P* = 0.006).

**Conclusion:**

Frailty is highly prevalent in older hemodialysis patients and closely correlated with increased mortality. Early frailty screening and risk stratification are essential for older dialysis patients and individuals at high risk of adverse clinical events. This study highlights the clinical value of early identification and standardized management of frailty, which can provide evidence for clinical diagnosis and treatment in this vulnerable population.

## 1 Introduction

Chronic kidney disease (CKD) is highly prevalent among older adults. With disease progression to end-stage renal disease, most patients eventually require renal replacement therapy. In China, the overall prevalence of this condition is approximately 1 per 100,000 population (1). Hemodialysis is the predominant form of renal replacement therapy. Frailty, a common geriatric syndrome, is characterized by diminished strength, endurance, and functionality. Among patients receiving maintenance hemodialysis (MHD), older adults are particularly vulnerable to frailty, mainly attributed to uremic toxin accumulation and nutritional depletion related to long-term dialysis (2). Accumulating evidence has confirmed that frailty serves as an independent risk factor for adverse clinical outcomes in MHD patients (3), with this association being more pronounced among older adult dialysis patients. A retrospective study by Okir et al. demonstrated that the frail group exhibited significantly higher readmission and mortality rates within 2 years compared to the non-frail group (4). This condition not only aggravates the disease burden but also substantially elevates the care burden. Nevertheless, prospective studies focusing on the prognostic impact of frailty among dialysis patients remain scarce in China. Emerging evidence indicates that interventions effective for frail older adults may also improve clinical outcomes in frail dialysis populations. This prospective cohort study explored the association between frailty and long-term survival among hemodialysis patients and analyzed the relevant risk factors for frailty. Notably, we further compared the differential impacts of frailty on survival across age subgroups of older dialysis patients. The findings may provide evidence for the prevention of frailty and the formulation of targeted interventions to ameliorate frailty status in this population.

## 2 Materials and methods

### 2.1 Population

A total of 397 patients receiving maintenance hemodialysis in our hospital from June to July 2022 were enrolled, and all participants underwent frailty assessment. The inclusion criteria were as follows: (1) aged ≥ 18 years (individuals aged over 60 years were defined as the older adults in this study) with a dialysis vintage of no less than 3 months; (2) voluntary participation in the study with signed written informed consent. Exclusion criteria: (1) patients with cognitive impairment; (2) patients hospitalized for treatment of various diseases; (3) patients with malignant diseases such as tumors and a life expectancy of less than 3 months.

All participants completed the 24-month follow-up, and survival outcomes were observed and recorded. This study was approved by the Ethics Committee of our hospital.

### 2.2 Data collection

A standardized comprehensive questionnaire was used to collect general demographic information, including age, gender, marital status, monthly income and residential location. Relevant clinical data were also extracted, such as dialysis vintage, dialysis frequency, vascular access type and blood flow parameters. The average weekly blood flow was analyzed exclusively, with flow reductions caused by vascular access complications excluded. Moreover, the questionnaire recorded body mass index (BMI), ultrafiltration rate, and the occurrences of adverse clinical events within the previous month, including fractures, cardiovascular and cerebrovascular events, as well as outpatient, emergency and hospitalization encounters.

Frailty was assessed using the simple FRAIL scale proposed by the International Association of Nutrition and Aging, which includes five items represented by the acronym “FRAIL” (Fatigue, Resistance, Ambulation, Illness, and Loss of weight). According to the scoring criteria, participants with a total score > 2 were classified as frail, those with a score of 1–2 were defined as pre-frail (an intermediate transitional state), and a score of 0 indicated a robust, non-frail status. The Cronbach’s aαcoefficient of the scale was 0.820. With informed consent obtained from all participants, face-to-face questionnaire interviews were conducted by trained research nurses on routine dialysis days. Ultimately, 391 valid questionnaires were collected, yielding an effective response rate of 98.5%.

### 2.3 Statistical

Analyses were conducted using SPSS 20.0, with the data entered in pairs. Descriptive statistics for categorical data are expressed as frequencies and percentages (%). For continuous data, normally distributed variables were reported as mean ± standard deviation (x ± s), whereas non-normally distributed variables were presented as medians (P25, P75). Comparative analyses between groups were performed using *t*-tests, Mann–Whitney U-tests, chi-square tests, and ANOVA. Factors influencing frailty were examined through multivariate theoretical logistic regression, and Cox regression was utilized for mortality risk analysis and the generation of survival curves. A significance level of α = 0.05 was applied, with two-sided *p*-values of less than 0.05, indicating statistical significance.

## 3 Results

### 3.1 Patient demographics

**FIGURE 1:**
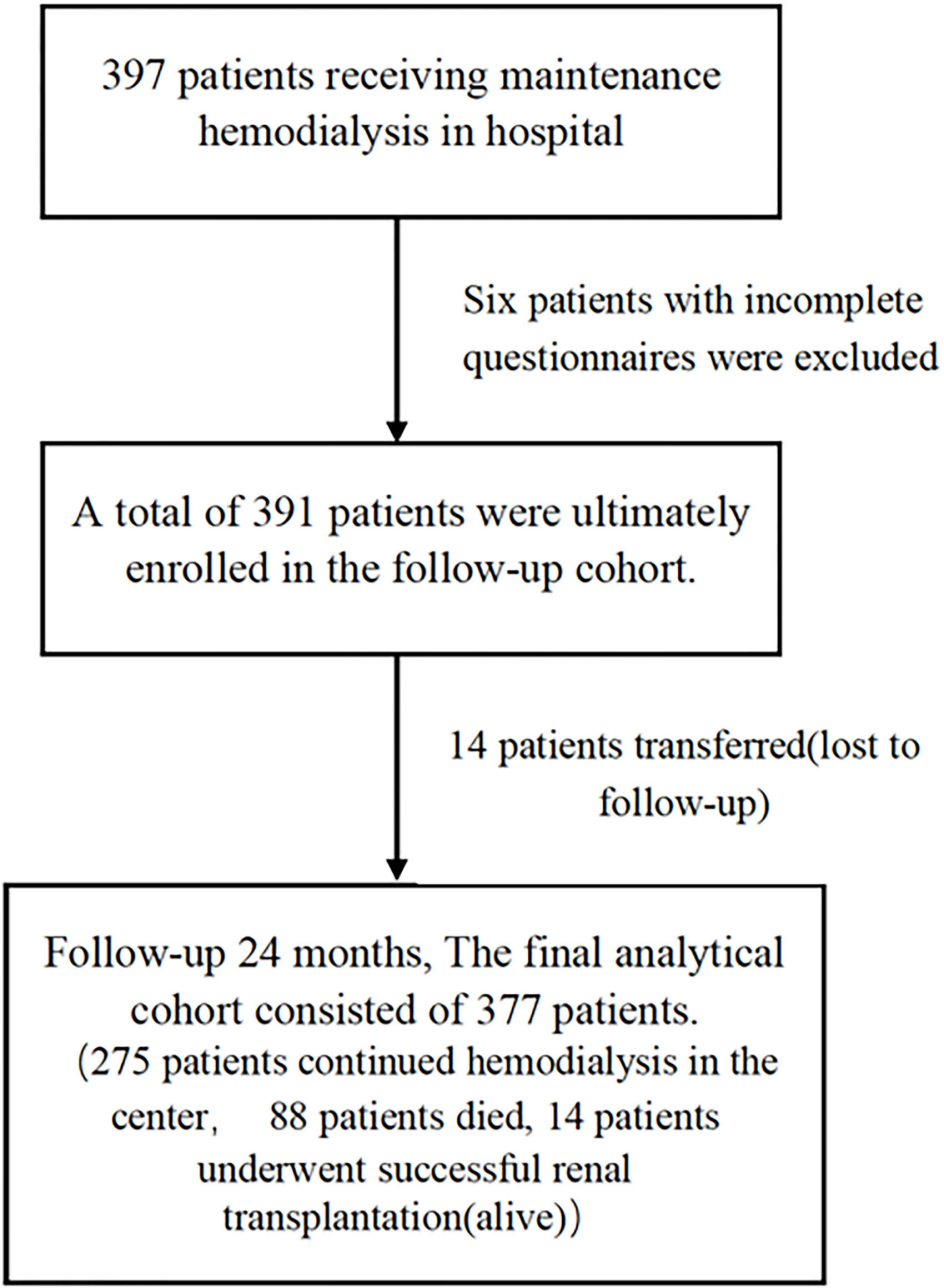
Patient exclusion flow chart.

Figure 1 provides a detailed overview of the cohort inclusion and exclusion criteria. The cohort consisted of 222 male patients, representing 56.9% of the sample, with a mean age of 61 ± 13.9 years and the median dialysis vintage was 4 (2.0, 8.5) years. Patients aged ≥ 60 years accounted for 57.5% of the total sample. The mean body mass index (BMI) was 23.8 ± 4.4 kg/m^2^, and the median ultrafiltration rate was 3.8% ± 1.3%. In total, 91.3% of participants received regular hemodialysis three times weekly, and 98.9% completed a 4-h dialysis session each time, which conformed to the standard dialysis regimen. Laboratory indicators showed a mean serum albumin level of 40.2 ± 3.2 g/L, mean hemoglobin of 111.9 ± 15.6 g/L, and mean total leukocyte count of 6.6 ± 1.9 × 10^9^/L. Detailed baseline demographic and clinical characteristics are summarized in Table 1.

**TABLE 1:** Demographic and clinical characteristics of patients undergoing MHD (*n* = 391).

Parameters	Variable	Patients (n)	Proportion (%)
Sex	Male	222	56.8
	Female	169	43.2
Age level	< 60 (years)	166	42.5
	= 60 (years)	225	57.5
Education	Elementary school	33	8.40
	Middle school	179	45.8
	College/University	159	40.7
	Postgraduate	20	5.10
Marital status	In marriage	237	60.6
	Unmarriage	128	32.7
	Divorced widowed	26	6.70
Residence status	Living alone	39	10.0
	Live with family/Nanny	352	90.0
Monthly income (RMB)	<2,000	48	12.3
	2,000–4,000	112	28.6
	4,000–6,000	118	30.3
	>6,000	113	28.9
Primary disease	Glomerulonephritis	100	25.6
	Hypertension	98	25.1
	Diabetes	92	23.5
	Other (immune disease, drug-induced injury, polycystic kidney disease)	101	25.8
Blood flow of HD (mL/min)	< 200	55	14.1
	200∼230	138	35.3
	230∼260	186	47.6
	> 260	12	3.1
Vascular access	Autogenous arteriovenous fistula	322	82.4
	Artificial vascular fistula	42	10.7
	Central venous catheter	27	6.9
Clinical events	Not occurring	350	89.5
	Occurs	41	10.5

HD, hemodialysis. In this table shows the basic information of all included investigators, the primary disease of dialysis treatment, such as the blood flow during treatment. Clinical events refer to events that occur within the past month, included fractures, cardiovascular and cerebrovascular incidents, and outpatient and emergency room hospitalizations within the past month.

### 3.2 Frailty condition

**FIGURE 2:**
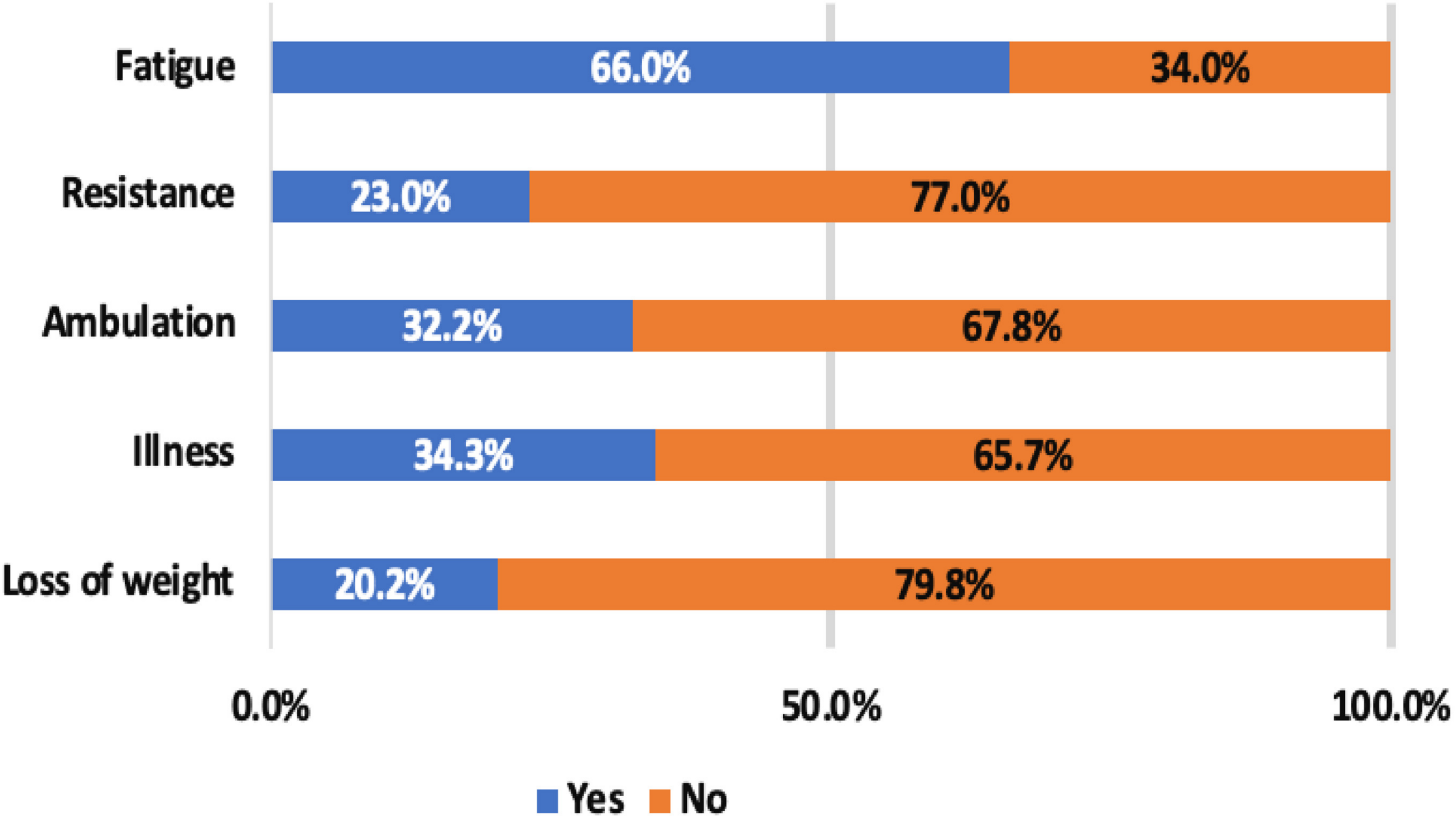
Frailty screening results for patients undergoing MHD. Figure 2 shows the results of the five dimensions of the FRAIL Frailty Scale, with fatigue being the most severe, with 66% of researchers answering yes. This was followed by 34.2% of patients with more than five diseases.

In the present study, the median total frailty score of all participants, assessed via the FRAIL Scale, was 2 (1, 3). Among these participants, 52.7% (*n* = 206) were classified as pre-frail, 28.6% (*n* = 112) were diagnosed with frailty syndrome (frail), and 18.7% (*n* = 73) were identified as non-frail. The distribution of scores across the five dimensions of the FRAIL Scale—Fatigue, Resistance (ability to climb one flight of stairs unassisted), Ambulation (ability to walk 100 meters), Illness (comorbidity with ≥ 5 chronic diseases), and Weight loss—is illustrated in Figure 2. Among these dimensions, Fatigue exhibited the highest prevalence, followed by Illness.

### 3.3 Frailty impact factors

Significant differences in age stratification (>60 years vs. ≤ 60 years), serum albumin, dialysis blood flow and the incidence of adverse clinical events were detected across groups with different frailty statuses. Patients in the frail group were older, accompanied by lower dialysis blood flow and decreased serum albumin levels. Among participants aged over 60 years, 85.3% were categorized as pre-frail or frailty syndrome. Increased frailty severity was correlated with a higher incidence of clinical adverse events, while no significant differences were found in other indicators. Relevant detailed data are shown in Table 2.

**TABLE 2:** Results of univariate analysis of the frailty status of patients undergoing MHD (*n* = 391).

Parameters	Variable	Non frailty (*n* = 73)	Pre-frail (*n* = 206)	Frailty syndrome (*n* = 112)	Test	*P*-value
Sex n(%)	Male	42(57.5)	125(60.7)	55(49.1)	χ^2^ = 3.98	0.123
	Female	31(42.5)	81(39.3)	57(50.9)		
Age group n(%)	< 60 (years)	40(54.8)	101(49.0)	25(22.3)	χ^2^ = 36.8	< 0.001
	> = 60 (years)	33(45.2)	105(41.7)	87(76.7)		
Education n(%)	Elementary school	5(6.8)	11(5.4)	17(15.2)	f = 11.161	0.084
	Middle school	30(41.1)	97(47.1)	52(46.4)		
	College/University	34(46.6)	87(42.2)	38(33.9)		
	Postgraduate	4(5.5)	11(5.3)	5(4.5)		
Marriage n(%)	In married	36(49.3)	127(61.6)	74(65.2)	χ^2^ = 6.94	0.139
	unmarried	33(45.2)	64(31.1)	31(28.4)		
	Divorced / widowed	4(5.5)	15(7.3)	7(6.4)		
Residence status n(%)	Living alone	7(9.6)	20(9.7)	12(10.7)	χ^2^ = 0.10	0.953
	Live with family/Nanny	66(90.4)	186(90.3)	100(89.3)		
Monthly income (RMB) n(%)	< 2000	7(9.6)	29(14.1)	13(11.6)	χ^2^ = 10.35	0.110
	2,000–4,000	27(37.0)	55(26.7)	30(26.8)		
	4,000–6,000	16(21.9)	58(28.1)	44(39.3)		
	> 6000	23(31.5)	64(31.1)	25(22.3)		
Primary disease n(%)	Glomerulonephritis	20(27.4)	56(27.2)	24(21.4)	χ^2^ = 3.54	0.738
	Hypertension	19(26.0)	48(23.3)	31(27.7)		
	Diabetes	13(17.8)	49(23.8)	30(26.8)		
	Other (immune disease, drug-induced injury, polycystic kidney disease)	21(28.8)	53(25.7)	27(24.1)		
Vascular access n(%)	Autogenous arteriovenous fistula	62(84.9)	172(83.5)	88(78.6)	χ^2^ = 3.71	0.447
	Artificial vascular fistula	7(9.6)	23(11.2)	12(10.7)		
	Central venous catheter	4(5.5)	11(5.3)	12(10.7)		
Blood flow of HD (ml/min) n(%)	< 200	4(5.5)	21(10.2)	30(26.8)	f = 53.09	< 0.001
	200∼230	20(27.4)	64(31.1)	54(48.2)		
	> 230∼260	43(58.9)	116(56.3)	27(24.1)		
	> 260	6(8.2)	5(2.4)	1(0.9)		
Clinical events n(%)	Not occurring	71(97.3)	186(90.3)	93(83.0)	χ^2^ = 9.81	0.007
	occurs	2(2.7)	20(9.7)	19(17.0)		
Age(years), (mean ± SD)	57.7 ± 13.0	58.4 ± 13.0	68.0 ± 13.8	*F* = 21.80	< 0.001
Years of dialysis median [25–75p]		4.0(2.0, 9.0)	4.0(2.0, 8.0)	4.5(3, 9)	*H* = 4.21	0.122
BMI (mean ± SD)		24.0 ± 4.2	24.0 ± 4.3	23.1 ± 4.6	*F* = 1.92	0.148
Ultrafiltration rate(%)(mean ± SD)		3.73 ± 1.46	3.92 ± 1.25	3.67 ± 1.15	*F* = 1.55	0.215
Hb(g/L) (mean ± SD)		110.4 ± 17.0	112.8 ± 15.3	111.2 ± 15.2	*F* = 0.74	0.476
Albumin(g/L) (mean ± SD)		41.2 ± 2.6	40.5 ± 3.3	38.9 ± 3.2	*F* = 14.33	< 0.001
WBC ( × 10^9^/L) (mean ± SD)		6.8 ± 1.9	6.8 ± 1.9	6.2 ± 1.9	*F* = 2.75	0.065

Hb, Hemoglobin; WBC, Total Leukocyte Count; f: Fisher test results. In this table shows a univariate analysis of frailty, among 225 elderly people aged > 60 years, 105 cases (46.7%) were screened with pre-frailty and 87 (38.7%) were screened for frailty syndrome. more frail patients choose low-flow dialysis; The incidence of clinical events is higher.

Multivariate logistic regression analysis was performed with frailty status designated as the dependent variable. All variables with a univariate analysis result of *P* < 0.1, including age, serum albumin, dialysis blood flow and clinical events, were enrolled into the regression model. Meanwhile, body mass index, hemoglobin and total leukocyte count were further incorporated into the model based on clinical evidence and previous literature, given their well-documented correlations with frailty.

The logistic regression outcomes indicated that adverse clinical events and decreased dialysis blood flow were independently correlated with frailty severity. Taking the non-frail status as the reference group, the occurrence of clinical events served as factor associated with pre-frailty (odds ratio (OR) = 4.88, 95% CI: 1.00, 23.75), *P* = 0.049) and frailty syndrome (OR = 5.90, 95% CI: 1.140, 30.51, *P* = 0.034). Lower dialysis blood flow is a clinical correlate of frailty. According to the regression models in Table 3, compared with higher dialysis blood flow ( > 260 mL/min), patients with lower blood flow settings (200–230 mL/min and 230–260 mL/min) were more likely to be categorized as pre-frail, with OR values of 4.26 (95% CI: 1.08–16.83) and 4.25 (95% CI: 1.15–15.65), respectively. The wide confidence intervals of these ORs indicated substantial instability of the regression results. Dialysis blood flow is prone to fluctuation and frequent adjustment during treatment due to multiple clinical factors, such as hypotension and poor vascular access function, together with limitations related to sample size. In addition, reduced serum albumin and advanced age were independent risk factors for frailty syndrome, with OR values of 0.83((95% CI: 0.74, 0.94) and 1.03(95% CI: 1.01, 1.07). Detailed data are summarized in Table 3.

**TABLE 3:** Multivariate regression analysis of frailty in patients undergoing MHD (N = 391).

Frailty status	Variable	*B*	Wald	Sig.	OR	95% CI for OR
Pre-frail	Intercept	1.78	0.45	0.502	0.92	(0.83, 1.01)
	Albumin (g/L)	-0.08	2.86	0.091		
	Age (years)	-0.01	0.70	0.403	0.99	(0.97, 1.01)
	BMI (kg/m^2^)	-0.01	0.15	0.702	0.988	(0.93, 1.05)
	HB(gı/L)	0.08	3.51	0.061	1.018	(0.99, 1.04)
	Blood flow < 200 mL/min*	1.57	3.08	0.079	4.802	(0.83, 27.72)
	Blood flow 200∼230 mL/min*	1.45	4.26	0.039	4.256	(1.08, 16.83)
	Blood flow 230∼260 mL/min*	1.45	4.72	0.03	4.247	(1.15, 15.65)
	Clinical events^†^	1.59	3.86	0.049	4.884	(1.00, 23.75)
Frailty syndrome	Intercept	2.30	0.48	0.487	0.834	(0.74, 0.94)
	Albumin (g/L)	-0.18	8.87	0.003		
	Age (years)	0.03	5.51	0.019	1.035	(1.01, 1.07)
	BMI (kg/m^2^)	-0.05	1.40	0.237	0.955	(0.89, 1.03)
	HB(gı/L)	0.02	3.12	0.078	1.021	(0.99, 1.04)
	Blood flow < 200 mL/min*	2.42	3.56	0.059	11.201	(0.91, 137.75)
	Blood flow 200∼230 mL/min*	2.33	4.01	0.045	10.244	(1.05, 99.95)
	Blood flow 230∼260 mL/min*	1.31	1.30	0.254	3.72	(0.39, 35.56)
	Clinical events^†^	1.77	4.47	0.034	5.895	(1.140, 30.51)

OR, odds ratio. *Reference: Blood flow > 260 mL/min;

† Reference: no clinical events. Multiple logistic regression analysis showed that the occurrence of clinical events was an associated factor for the occurrence of pre-frailty (OR = 4.89) and frailty syndrome (OR = 5.90). Patients with low blood flow settings are more likely to have comorbid frailty (OR 200–230 ml/min = 4.26 and OR 230–260 ml/min = 4.25) (OR 200–230 ml/min = 10.24). Low albumin and advanced age are risk factors for asthenic syndrome.

### 3.4 Univariate analysis of survival

All 391 patients were followed up for 24 months. During the follow-up period, 275 patients continued hemodialysis at the study center, 14 patients underwent renal transplantation, and 14 patients were transferred to other dialysis centers. A total of 88 patients died, including 50 males (accounting for 56.8%). The deceased patients had a mean age of 70.7 ± 11.2 years and a median dialysis vintage of 6 (3, 9) years.

The composition of primary diseases leading to dialysis in the deceased patients was as follows: glomerulonephritis (14.8%), hypertension-induced nephropathy (31.8%), diabetic nephropathy (33.0%), and other etiologies (immune disorders, drug-induced renal injury, polycystic kidney disease, etc.) (20.5%). The median follow-up duration for the deceased patients was 14 (8, 19) months, among whom 39 died within 12 months of follow-up.

To explore the relationship between frailty and long-term survival, Cox regression analysis was performed to evaluate the 12-month and 24-month survival outcomes of enrolled patients. Age and primary renal diseases closely associated with mortality were included as confounding variables, and multiple regression models were established for comparative analysis.

The results demonstrated that elevated frailty status significantly increased the all-cause mortality risk at both 12-month and 24-month follow-up (Tables 4, 5). Specifically, patients in the frail group presented a markedly higher 12-month mortality risk than the non-frail group, with a hazard ratio (HR) of 5.95(95% CI: 1.79, 19.76) (Model 1 in Table 4). After further adjustment for confounding factors such as age and primary disease, the associative effect of frailty was attenuated in the regression models, with decreased HR and *P*-values close to the statistical threshold (*P* = 0.050 *P* ≈ 0.049) (Model 2 and Model 3 in Table 4). Nevertheless, increased frailty severity remained positively associated with elevated mortality risk.

**TABLE 4:** Cox regression analysis of 12-month survival in patients undergoing MHD (*N* = 377).

Model	Variable	B	SE	Wald	Sig.	HR	95.0% CI for HR	-2 Log likelihood	Chi-square	Sig.
Model(1)	Frailty status	0.36	0.65	21.04	< 0.001	1.43	(0.40, 5.05)	436.19	25.51	< 0.001
	Pre-frail*			0.30	0.583					
	Frailty syndrome*	1.78	0.61	8.48	0.004	5.95	(1.79, 19.76)			
Model(2)	Frailty status	0.31	0.65	7.56	0.023	1.37	(0.39, 4.85)	416.60	42.61	< 0.001
	Pre-frail*			0.24	0.627					
	Frailty syndrome*	1.20	0.63	3.69	0.050	3.33	(0.98, 11.37)			
	Age	0.07	0.02	15.97	< 0.001	1.07	(1.03, 1.10)			
Model(3)	Frailty status	0.35	0.65	7.58	0.023	1.42	(0.40, 5.02)	415.57	43.30	< 0.001
	Pre-frail*			0.29	0.592					
	Frailty syndrome*	1.23	0.63	3.87	0.049	3.42	(1.00, 11.68)			
	Age	0.07	0.02	16.43	< 0.001	1.07	(1.04, 1.10)			
	Primary disease	-0.15	0.15	1.03	0.311	0.86	(0.64, 1.15)			

HR, hazard ratio.

*Reference: Non-frail patients. COX regression analysis showed that the risk of death within 12 months was significantly higher in the frailty syndrome group than in the non-frail group, HR = 5.95. After the inclusion of two factors, age and primary disease, the risk coefficient of frailty was reduced, HR = 3.33 and HR = 3.42.

**TABLE 5:** Cox regression analysis of 24-month survival in patients undergoing MHD (*N* = 377).

Model	Variable	*B*	SE	Wald	Sig.	HR	95.0% CI for HR	-2 Log Likelihood	Chi-square	Sig.
Model(1)	Frailty status	0.41	0.42	39.87	< 0.001	1.50	(0.66, 3.43)	924.32	47.12	< 0.001
	Pre-frail*			0.94	0.333					
	Frailty syndrome*	1.71	0.41	17.84	< 0.001	5.54	(2.50, 12.26)			
Model(2)	Frailty status	0.36	0.42	13.57	0.001	1.43	(0.62, 3.26)	882.89	84.13	< 0.001
	Pre-frail*			0.71	0.4					
	Frailty syndrome*	1.14	0.42	7.51	0.006	3.12	(1.38, 7.05)			
	Age	0.06	0.01	34.22	< 0.001	1.07	(1.04, 1.09)			
Model(3)	Frailty status	0.36	0.42	13.66	0.001	1.44	(0.63, 3.29)	882.56	84.21	< 0.001
	Pre-frail*			0.74	0.388					
	Frailty syndrome*	1.15	0.42	7.64	0.006	3.15	(1.40, 7.13)			
	Age	0.07	0.01	34.32	< 0.001	1.07	(1.04, 1.09)			
	Primary disease	-0.06	0.10	0.33	0.567	0.95	(0.78, 1.15)			

HR, hazard ratio. *Reference: Non-frail patients. COX regression analysis showed that the risk of death within 24 months was significantly higher in the frailty syndrome group than in the non-frail group, HR = 5.54. After the inclusion of two factors, age and primary disease, the risk coefficient of frailty was reduced, HR = 3.12 and HR = 3.15.

Consistently, frailty remained an associated factor for 24-month mortality. Relative to the non-frail population, the frail group had an HR of 5.54 [95% CI (2.50, 12.26)] (Model 1 in Table 5). Notably, this significant association persisted after sequential adjustment for age and primary disease, with adjusted HRs of 3.12(95% CI: 1.38, 7.05) (Model 2 in Table 5) and 3.15(95% CI: 1.40, 7.13) (Model 3 in Table 5).

With the non-frail group as the reference, comparison of regression models at different follow-up time points demonstrated that the frail group exhibited a higher hazard ratio (HR) at the 12-month follow-up than at the 24-month follow-up, indicating that frailty exerts a more prominent impact on short-term mortality. No significant difference in survival outcomes was observed between the pre-frail and non-frail groups in this model.

**FIGURE 3:**
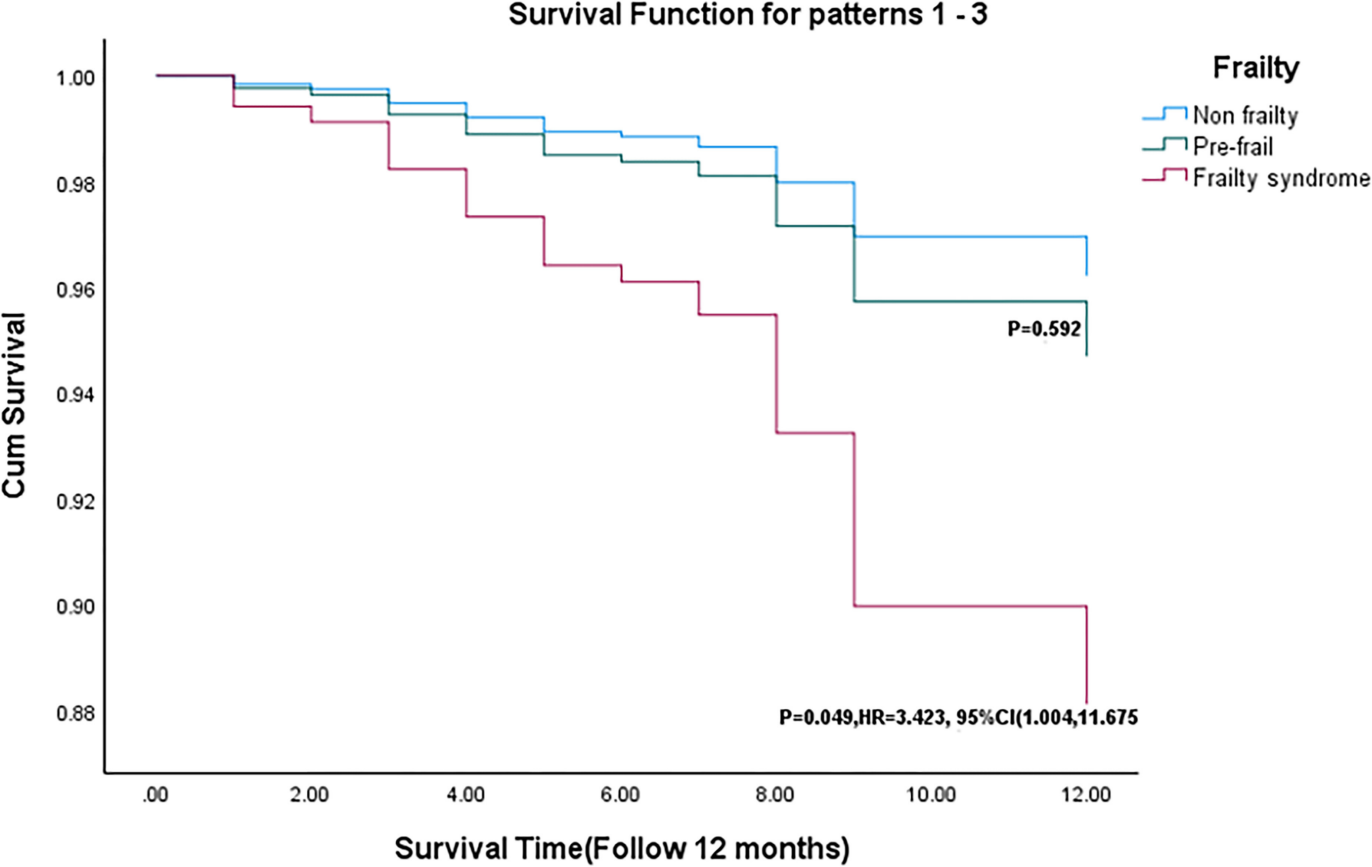
Survival analysis curve of frailty degree of 12-month. Taking the non-frail group as a reference, there was no statistically significant difference in survival between the pre-frail group and the non-frail group. The frailty group had a more significant effect on mortality within 12 months, with HR = 3.423.

**FIGURE 4:**
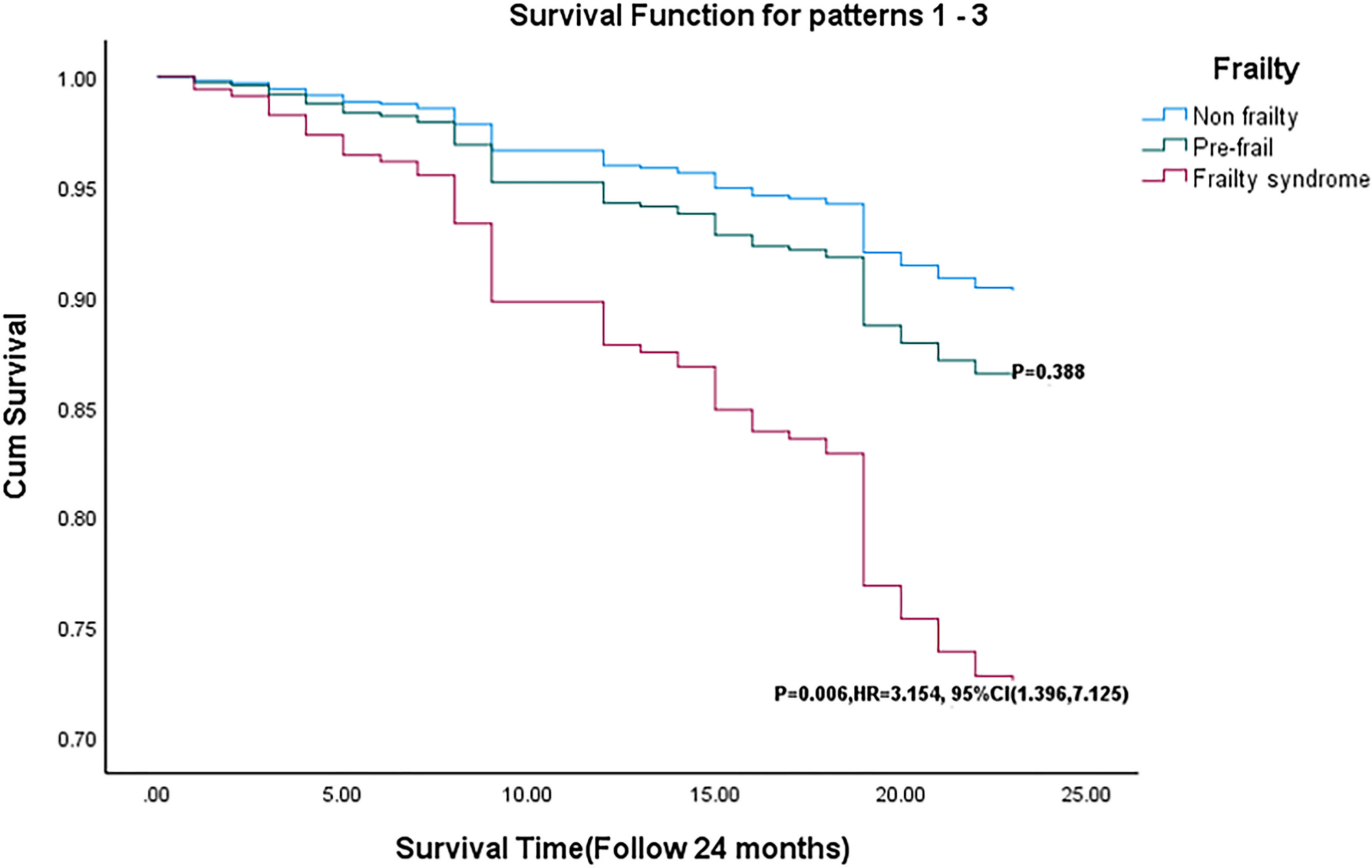
Survival analysis curve of frailty degree of 24-month. Note: Taking the non-frail group as a reference, there was no statistically significant difference in survival between the pre-frail group and the non-frail group. The frailty group had a more significant effect on mortality within 24 months, with HR = 3.154.

Across all regression models, increased age was an risk factor for mortality. Each 1-year increment in age was associated with a 7% increase in the risk of death (HR = 1.07, 95% CI: 1.04, 1.09). In addition, primary renal disease showed no significant correlation with long-term survival. Survival curves stratified by frailty status were plotted and presented in Figure 3, 4.

Age was an independent determinant of mortality. Of all decedents during the 24-month follow-up, 74 cases (84.0%) were aged over 60 years, reflecting a predominance of elderly individuals. To further explore age-related differences, subgroup analysis was performed with 70 years as the cut-off value. In the elderly subgroup aged ≥ 70 years (*n* = 115), 54 patients died; among 102 patients aged 60–69 years, 20 deaths occurred.

With frailty syndrome set as the reference category, frailty indicators were incorporated into the Cox regression model. The results showed that both non-frail and pre-frail statuses were associated with a significantly lower mortality risk, with HRs of 0.11(95% CI: 0.01, 0.87) and 0.30(95% CI: 0.12, 0.74), respectively. In the subgroup of patients aged ≥ 70 years, pre-frail individuals also presented a reduced mortality risk relative to those with frailty syndrome (HR = 0.41,95% CI: 0.22, 0.78). Detailed subgroup analysis data are summarized in Table 6.

**TABLE 6:** COX regression analysis of 24-month survival in patients undergoing MHD stratified by age (*N* = 217).

Group	Parameters	B	SE	Wald	Sig.	HR	95.0% CI for HR	-2 Log Likelihood	Chi-square	Sig.
Age >= 60 and < 70 (*n* = 102)	Frailty status	-2.03	1.05	9.57	0.008	0.11	(0.01, 0.87)	171.00	11.70	0.003
	Non-frailty*			4.45	0.035					
	Pre-Frail*	-1.22	0.47	6.86	0.009	0.30	(0.12, 0.74)			
Age >= 70 (*n* = 115)	Frailty status	-0.48	0.44	7.70	0.021	0.62	(0.26, 1.49)	458.05	8.17	0.017
	Non-frailty*			1.14	0.285					
	Pre-Frail*	-0.89	0.33	7.44	0.006	0.41	(0.22, 0.78)			

HR, hazard ratio.

*Reference: patients with frailty syndrome. In the age group ≥ 60 to 70 years, using frailty syndrome < as a reference, patients without frailty and pre-frailty had a lower risk of death at 24 months, HR = 0.11 and HR = 0.30 In the 70-year-old ≥ subgroup, the risk of death in the pre-frailty period was lower than in patients with asthenic syndrome, HR = 0.41.

## 4 Discussion

Frailty has been defined as “an age-related state characterized by decreased strength and physiological function that increases individual vulnerability, leading to increased dependence, vulnerability, and mortality” (5). In 2019 and 2020, the International Conference of Frailty and Sarcopenia Research (ICFSR) developed the related guidelines “Guidelines for Screening and Management of Frail Patients in Primary Care” (6), emphasizing that older adults should be screened regularly for the management of debility. The FRAIL scale adopted in the present study is recommended by the 2020 ICFSR guidelines as a rapid screening instrument for primary care. It has been well validated to predict multiple adverse clinical outcomes, including disability and all-cause mortality (7). Additionally, accumulating evidence indicates that this scale presents a favorable correlation with dialysis-associated complications in Chinese populations (8), rendering it highly applicable for patients undergoing maintenance dialysis.

### 4.1 Frailty is prevalent in MHD patients

Declining renal function increases the risk of frailty in patients, with chronic inflammation and malnutrition serving as crucial contributing factors (2, 9, 10). Accordingly, the prevalence of frailty among dialysis patients is markedly higher than that in the general older adult population and individuals with other chronic diseases (11). In this cohort, only 18.7% of participants were non-frail, while 52.7 and 28.6% were categorized as pre-frail and having frailty syndrome, respectively.

A meta-analysis indicated that frailty prevalence among Chinese MHD patients was 37.4% [95% CI (30.3%–44.5%)] (12), with 30.7% reported using the FRAIL scale, consistent with this study. These variations are attributed to geographical differences among subjects and differences in investigation timing, age, and disease severity. Jung et al. (13) demonstrated that pre-frail individuals have a high risk of progressing to overt frailty within 3–5 years. A considerable proportion of pre-frail patients were identified in this study, which warrants close attention from clinical staff. The World Health Organization’s Integrated Care for Older People (ICOPE) initiative targets pre-frail and frail individuals, advocating early intervention to slow or even reverse the progression of frailty.

Endorsed by the ICFSR guidelines (6), this strategy is well suited for primary care settings. It highlights the importance of early frailty screening and individualized intervention to reduce frailty burden and improve long-term clinical outcomes, which further explains the core objective and clinical significance of the current study.

Both natural and pathological factors influence frailty changes, with vitamin D, serum proteins, and hemoglobin identified as contributors in previous studies (14). In this study, regression analysis revealed that serum albumin levels were negatively correlated with frailty scale scores, which was consistent with the findings of previous studies (8). Most current studies on frailty among maintenance hemodialysis (MHD) patients have incorporated limited dialysis-specific indicators. This study examined the effects of vascular access, blood flow, ultrafiltration rate, and dialysis-related clinical events observable during treatment. The study found frailty was negatively correlated with blood flow in patients with lower tolerable blood flow. Blood flow refers to the average flow over three dialysis sessions (1 week) in patients with adequate vascular access. Dialysis blood flow was comprehensively determined by clinicians based on individual conditions, including age, blood pressure, body mass index, type of vascular access, dialyzer specification and dialysis adequacy and is adjusted for patient comfort without reported discomfort. Restricted by baseline characteristics such as body weight and body mass index, routine clinical dialysis blood flow is generally set at 200–260 mL/min. For older adults with central venous catheter access, blood flow is commonly maintained above 180 mL/min. In male patients with a high body mass index and favorable cardiac function, blood flow can be adjusted to 280 mL/min, and such patients account for less than 10% of the overall cohort.

Indicators of overall dialysis indicators for the center’s patients are favorable, are monitored by the quality control center. Frailty manifests as progressive physical function decline and is jointly affected by physiological, pathological and social factors. Current research on dialysis-related complications remains inadequate. This study collected dialysis-associated clinical events occurring within 1 month before patient assessment, focusing on fractures, cardiovascular diseases, and hospitalizations. Considering the adverse impacts of acute illnesses on physical status, patients receiving active treatment were excluded, and only stable participants who had resumed regular dialysis were enrolled. The present study found that even when patients’ biochemical indicators remained within stable ranges after the occurrence of the above clinical events, these events could impair physical function, exacerbate frailty severity, and further reduce quality of life. The findings suggest that clinical attention should be paid to the early screening and comprehensive management of pathological frailty. In routine clinical practice, patients’ conditions change dynamically and frequently; repeated scale screening and multi-index biochemical evaluation are of low feasibility. Dialysis blood flow and specific clinical events can be easily observed by clinical staff and are closely correlated with patients’ subjective health outcomes. This provides a reasonable theoretical basis for our study and supports the in-depth exploration of dialysis-related clinical factors.

### 4.2 Frailty significantly affects long-term survival

Recent years have seen increasing focus on frailty status of patients undergoing maintenance hemodialysis (MHD) in China, with a gradual increase in related studies. Nevertheless, there is a lack of long-term follow-up research on frailty’s impact on patient survival. Adverse outcomes of frailty, including mortality, hospitalization, and other negative events, warrant attention. However, current research on this topic remains insufficient.

The primary objective of examining frailty is to assess prognosis and quality of life in dialysis patients. The 24-month follow-up results of this study demonstrated that multivariate regression models confirmed severe frailty was independently associated with elevated mortality risk in maintenance dialysis patients. At the 12-month follow-up, patients with frailty syndrome exhibited a 4.9-fold higher mortality risk relative to non-frail individuals (HR = 5.95). After adjusting for age and primary renal disease, the mortality risk remained significantly elevated (HR = 3.42). Limited by the single-center design and relatively small sample size, the corresponding P value was 0.049, indicating potential instability of this statistical model. In Model 2 (Table 4), adjusted only for age, the HR of frailty syndrome was 3.33 with a *P* value of 0.05, suggesting sample size influence, requiring verification in larger multicenter studies.

Results from the three models in Table 6 indicate frailty’s impact on survival at 12 months was consistent. Correspondingly, prognostic models were established for the 24-month follow-up outcome. In Model 1, including only frailty-related variables mortality risk for frailty syndrome increased 4.5 times compared to patients without debilitating conditions (HR = 5.54). In Models 2 and Model 3 (shown in Table 6), HR values for age and post-primary frailty syndrome decreased, yet mortality risk remained three times higher than those without frailty, with statistical significance (p < 0.001). The model showed no significance in pre-frailty versus non-frailty stage. Further multicenter studies with expanded sample sizes are required to verify the generalizability of the present findings. Survival curves stratified by different frailty stages also intuitively reflected the above trends.

Frailty is associated with elevated morbidity and mortality (8, 9, 15–17). McAdams-DeMarco et al. (18) found that frailty was independently associated with mortality among hemodialysis patients (RR = 2.60, 95% CI: 1.04–6.49) and reported a 1.43-fold increase in hospitalization risk. Lee et al. (19) studied 1658 dialysis patients over 17.1 months, finding a 2.4-fold increase in rehospitalization risk and a 3.05-fold increase in mortality risk among frail patients. These findings align with previous studies.

Older Age is a well-recognized risk factor for elevated mortality. In this study, patients in the mortality group were older, with a mean age of 70 ± 11.2 years. Regression analysis revealed that each 1-year increase in age was associated with a 7% rise in the risk of death. Similarly, a meta-analysis by Luo et al. (20) identified age as a risk factor with mortality among Chinese patients with MHD. Age stratification was performed to explore the 24-month association between frailty status and survival across different age subgroups. The mean age of deceased patients was 70.7 years, and only 14 decedents were younger than 60 years, suggesting that most deaths occurred in older individuals. Among patients aged 60–69 years, pre-frail subjects exhibited a 70% lower mortality risk (HR = 0.30, 95% CI: 0.12, 0.74) relative to patients with frailty syndrome, while non-frail patients had a 90% reduced mortality risk (HR = 0.11, 95% CI: 0.01, 0.87). In the subgroup aged over 70 years, pre-frail individuals showed a decrease in mortality risk (HR = 0.41, 95% CI: 0.22, 0.78) compared with frail counterparts. In the subgroup aged over 70 years, pre-frail patients exhibited a reduced mortality risk compared with frail individuals (HR = 0.41, 95% CI: 0.22–0.78). Given the limited number of non-frail cases (only 14 participants), this statistical model presents certain instability. Although no significant statistical difference was observed for non-frail patients within this age stratum, a positive correlation was still indicated. Further large-sample studies are warranted to validate these findings. In conclusion, all regression models verified that frailty is an influential factor for the long-term prognosis of Chinese patients undergoing maintenance hemodialysis. Especially in elderly dialysis patients, frailty is closely associated with an increased risk of all-cause mortality within 2 years.

This single-center study has several limitations. First, sarcopenia-related indicators, including calf circumference, mid-upper arm circumference, and skeletal muscle mass measured via bioelectrical impedance analysis, were not assessed in the enrolled patients. Further high-quality studies are needed to clarify the potential prognostic implications of frailty combined with muscle wasting in hemodialysis patients. Second, this study employed a single-center design with a limited sample size. To minimize baseline heterogeneity associated with severe comorbidities and acute illness, patients with cognitive impairment, acute hospitalization, or advanced malignancy were excluded in accordance with the study objectives. This selection bias may restrict the generalizability of the study findings and lead to an underestimation of the overall frailty burden in the dialysis population. Future well-designed, large-sample, multicenter controlled trials are warranted to comprehensively investigate the association between frailty and hospitalization events, particularly pre-terminal hospitalization. Third, core physical performance parameters, such as handgrip strength and gait speed, were not evaluated in older dialysis patients. In future research, key nutritional and inflammatory biomarkers routinely monitored in dialysis patients—including serum prealbumin and high-sensitivity C-reactive protein—should be incorporated. Assessment of these biomarkers will help unravel the underlying mechanisms linking protein-energy wasting, chronic low-grade inflammation, and frailty, as well as further elucidate their combined effects on long-term adverse outcomes in patients receiving maintenance hemodialysis.

In conclusion, frailty is highly prevalent among patients undergoing maintenance hemodialysis, and progressive frailty is significantly associated with poor long-term survival outcomes. Given the ongoing population aging and the rapid increase in the number of dialysis patients in China, early frailty screening and risk stratification should be actively implemented for elderly dialysis patients and high-risk individuals. Early identification of frailty can provide a reference for subsequent individualized clinical management. This study highlights the critical clinical significance of timely recognition and standardized management of frailty in older dialysis populations.

## Data Availability

The original contributions presented in this study are included in the article/supplementary material, further inquiries can be directed to the corresponding authors.
